# Anomalies in Long-Crack Propagation at Low ΔK in Some Engineering Alloys

**DOI:** 10.3390/ma17246093

**Published:** 2024-12-13

**Authors:** Daniel Kujawski, Asuri K. Vasudevan

**Affiliations:** 1 Mechanical and Aerospace Engineering, Western Michigan University, Kalamazoo, MI 49008, USA; 2 TDA Inc., Falls Church, VA 22043, USA; akruva@gmail.com

**Keywords:** crack growth threshold, unusual FCG at low ΔK, damage-tolerant design, Marci effect

## Abstract

In this article, we discuss an unusual pattern in long-crack behavior at low stress intensity factor ranges ΔK (below ΔK_th_), characterized by an initial dip, followed by a plateau, and then an acceleration in fatigue crack growth (FCG) rate. This unanticipated FCG behavior was first observed experimentally in the IMI 834 alloy and reported by Marci in 1996. Such an anomaly is only reported from experimental observation but cannot be understood or explained using the plasticity, roughness, or oxide-induced crack closure assumptions. It also has not been fully explained through either metallurgical analysis or failure mode investigation. The established application of fracture mechanics to the FCG rate (da/dN) assumes that the FCG rate decreases with decreasing ΔK towards the threshold of ΔKth with (da/dN) ≤ 10^−7^ mm/cycle. Yet, some materials exhibit a lack of ΔK threshold dependence for long cracks when tested using constant-K_max_ or constant-R-ratio testing. An understanding of this anomaly and the related physics poses a scientific challenge. It is also relevant to predict the safe service life of structures subjected to high-frequency and low-amplitude vibrating loads. Here, we provide our interpretation and discuss the significant implications of this phenomenon in the context of damage-tolerant design.

## 1. Introduction

Typically, the behavior of engineering materials under cyclic loads is studied using two types of specimens: smooth specimens [1,2,3] and cracked fracture mechanics specimens [4,5]. Smooth specimens are tested until failure, while fracture mechanics specimens focus on studying fatigue crack growth (FCG) behavior.

Traditionally, fatigue life has been analyzed using stress- or strain-based approaches (S-N or ε-N curves) in terms of the number of cycles, N_f_ (or reversals 2N_f_), to failure, whereas the FCG rate (da/dN) has been analyzed in terms of the stress intensity factor range ΔK. As a result, these two approaches have been kept as separate domains in the fatigue and fracture mechanics fields. The S-N approach was developed for high cyclic fatigue (HCF), where loading is mostly elastic, whereas the ε_p_-N approach was developed for low cyclic fatigue (LCF) where plastic strain dominates [3].

Basquin’s Equation (1) is often used to describe S-N curves under the HCF regime:

(1) $$\sigma_{a}=\frac{∆\sigma }{2}=\sigma_{f}^{\prime }{\left(2N_{f}\right)}^{b}$$

and Manson–Coffin’s Equation (2) is used to describe ε_p_-N curves under LCF:

(2) $$\varepsilon_{a,p}=\varepsilon_{f}^{\prime }{\left(2N_{f}\right)}^{c}$$

To analyze both the LCF and HCF regimes, the total strain amplitude, *ε_a_* = *σ_a_*/*E* + *ε_a_*_,*p*_, is utilized:

(3) $$\varepsilon_{a}=\frac{\sigma_{f}^{\prime }}{E}{\left(2N_{f}\right)}^{b}+\varepsilon_{f}^{\prime }{\left(2N_{f}\right)}^{c}$$

where *E* is the Young’s modulus, $\sigma_{f}^{\prime }$ and $\varepsilon_{f}^{\prime }$ are strength and ductility coefficients, and b and c are strength and ductility exponents, respectively.

Usually, the R-ratio dependence on the S-N (or ε-N) curve is determined using so-called mean stress effect approaches such as those by Goodman and Morrow and the SWT as examples [3]. Testing at different R-ratios is more common for fatigue crack growth area where many R-ratio results are collected. A smooth tensile test is used in S-N (or ε-N) testing, while pre-cracked fracture mechanics samples are used in FCG testing.

For cracked body testing, the FCG rate (da/dN) is plotted versus the applied stress intensity range, ΔK, which is calculated as follows:

(4) $$∆K=F\;∆\sigma \sqrt{\pi \;a}$$

where *F* is a geometry factor (e.g., for a semi-circular surface crack, *F* = 0.728) and ‘a’ is the crack depth.

The threshold of FCG, $∆K_{th}$, is defined when (da/dN) = 10^−10^–10^−11^ m/cycle, which, from a practical viewpoint, is considered a non-propagating crack when applying ΔK ≤ ΔKth. During FCG testing, the independent/input variable is the applied stress intensity factor (SIF) range, ΔK, which is applied at a constant load R-ratio (= minimum load/maximum load) or at a constant maximum SIF, K_max_. The material response is represented by the dependent/output variable in terms of the FCG rate, da/dN.

### 1.1. Fatigue Testing of Smooth Specimens

**Figure 1:**
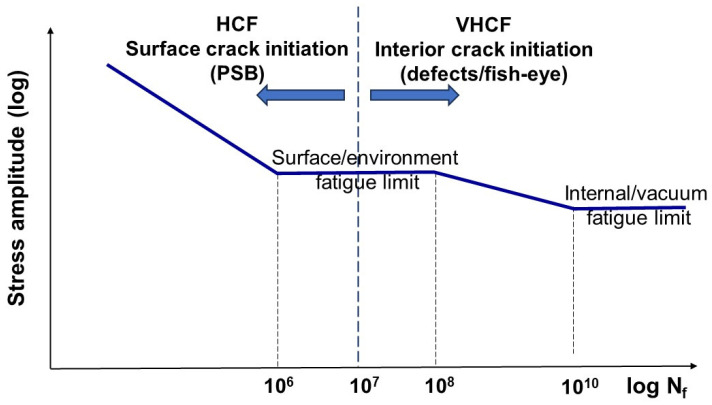
Illustration of a double bi-linear S-N fatigue life diagram related to the HCF and VHCF regions.

The number of cycles to failure, N_f_ ≤ 10^6^–10^7^, for smooth specimens is often characterized and studied using stress- or strain-based approaches, specifically through S-N or ε-N curves, respectively. The S-N representation is typically associated with high cycle fatigue (HCF) with N_f_ > 10^4^ cycles, whereas the ε-N representation is used for low cycle fatigue (LCF) with N_f_ ≤ 10^4^ cycles. HCF data are often represented with log–log or semi-log S-N curves (Figure 1). The horizontal or nearly horizontal part of the S-N curve represents the traditional or surface-related fatigue limit with lifetimes of 10^6^–10^7^ cycles. Hence, most traditional laboratory fatigue testing is limited to 10^7^–10^8^ cycles. However, there are many instances of failures at N_f_ > 10^8^ cycles under so-called very high cyclic fatigue (VHCF) or gigacycle fatigue regimes. Such VHCF failures may occur due to low-amplitude vibratory service loads, e.g., roller bearings, turbine blades, pumps, and axles. Therefore, with the introduction of ultrasonic testing equipment operating at 20 kHz, there has been significant interest in VHCF testing in the last 30 years. As a result, the first international conference solely dedicated to VHCF was organized in 1998 [6]. Since then, VHCF testing has become an important area of fatigue research, e.g., [7,8], where more references can be found. Figure 1 illustrates a double bi-linear fatigue life diagram corresponding to the HCF and VHCF regions.

For most low-strength engineering alloys, traditional HCF failure is associated with surface crack initiation at persistent slip bands (PSBs), whereas for high-strength alloys, inclusions or other internal defects are more commonly the source of initiation. Failures in the VHCF region, which are linked to internal impurities or material defects, often exhibit a fish-eye fracture appearance [6]. The surface fatigue limit for most low-strength steels is linked to the PSB threshold, where an initiated surface crack may stop at the grain boundary. On the other hand, the VHCF limit is related to internal defects with insufficient stress concentrations below the threshold for vacuum crack initiation.

### 1.2. Fatigue Testing of Cracked Specimens

**Figure 2:**
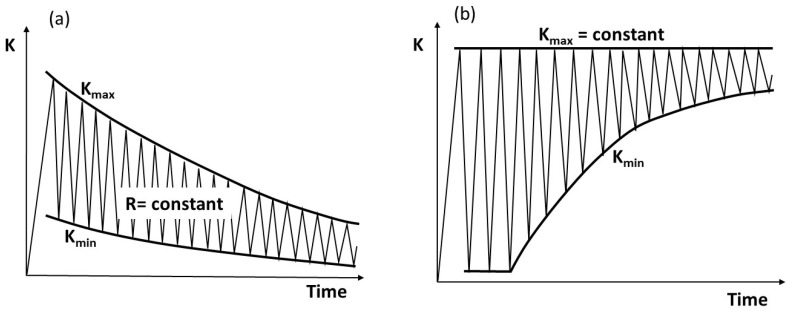
Illustration of the near-threshold loading procedures for (**a**) constant-R-ratio and (**b**) constant-K_max_ tests.

When a damage-tolerant approach was adopted in design, FCG testing was established [4,5]. Traditionally, FCG behavior near the threshold is tested by conducting R = constant or K_max_ = constant tests, as illustrated in Figure 2, while the FCG in the Paris region is usually studied with a constant load, i.e., increasing K, or under programmed load tests. A common notion is that, in general, the R-constant and the K_max_-constant test procedures for FCG testing are equivalent.

**Figure 3:**
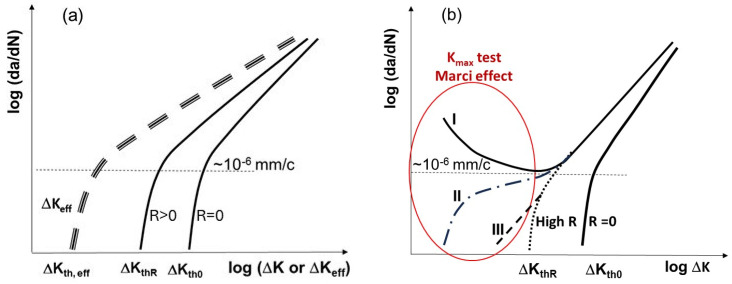
Graphical representation of FCG (da/dN) vs. ΔK, (**a**) showing ΔKth (or ΔKth_,eff_) and (**b**) denoting three types of unusual behavior (Marci effect) at ΔK < ΔKth under K_max_ =constant test.

More than 50 years ago, Paris and Erdogan [9] determined that the FCG rate (da/dN) could be correlated to the stress intensity factor range, ΔK, as follows:

(5) $$\frac{da}{dN}=C{∆K}^{m}$$

where *C* and *m* are material-related curve fitting parameters. This relationship is commonly known as the Paris law. It was found out later that when da/dN approaches approximately 10^−6^ mm/cycle, the Paris relation between log(da/dN) and log (ΔK) begins to diverge from Equation (1) when ΔK decreases and approaches the threshold ΔK_th_ (Figure 3a).

The solid curves in Figure 3a illustrate the da/dN behavior near the threshold and in the Paris region as a function of the applied ΔK at two different R-ratios. It is a common notion to define the threshold ΔKth as an FCG rate (da/dN) ≤ 10^−7^–10^−8^ mm/cycle [4,5]. Constant-R-ratio tests (R = P_min_/P_max_ = K_min_/K_max_) show that (da/dN)–ΔK curves (Figure 3a) shift to the left with increasing R and decrease monotonically with decreasing ΔK towards ΔK_th_. To account for the R-ratio shift in the FCG curves, Elber [10] proposed plotting da/dN versus ΔK_eff_ = K_max_ − K_cl_, where K_cl_ is the stress intensity factor corresponding to crack closure. The dashed curve in Figure 3a illustrates a single effective da/dN-ΔK_eff_ curve. The idea of collapsing all da/dN curves for different R-ratios into a single ‘effective curve’ proved useful for damage-tolerant design. The most commonly referenced mechanisms of crack closure are plasticity-induced, oxide-induced, and roughness-induced crack closure (PICC, OICC, and RICC). Recently, we examined and discussed how these closure mechanisms impact the shielding of the crack tip from the applied load [11,12,13]. The main conclusion was that environmental damage, rather than crack closure, primarily drives the observed R-ratio effects on FCG in air or other active environments. This is supported by the observation that in a vacuum, the R-ratio effect on FCG is either absent or minimal [14]. In current design practices, it is assumed that a long crack will not propagate in practical applications if ΔK is below ΔK_th_ (or ΔK_th,eff_). However, this assumption relies on the existence of ΔK_th_. Instead, numerous cases that have been reported in the literature over the past 30 years, such as [15,16,17,18,19,20,21,22,23,24,25,26,27,28], indicate unanticipated and unusual FCG behavior at low ΔK values below ΔK_th_ for long cracks. Based on cases reported in the literature, Figure 3b illustrates three types of unexpected FCG behavior: Type I, Type II, and Type III.

In VHCF tests, failures occur below the traditional fatigue limit. Similarly, unusual FCG behavior has been experimentally observed for some alloys at ΔK values below ΔK_th_. This is an important consideration for vibrating or rotating components subjected to high K_max_. Such unusual FCG behavior is known as the “Marci effect”. Interestingly, the Marci effect was observed and reported in FCG tests but was often dismissed as an unanticipated anomaly because the ΔK_eff_ approach could not explain it. The Marci effect at low ΔK values below the conventional ΔK_th_ is a real physical phenomenon that presents challenges for designers in VHCF applications.

In this article, the Marci effect, which is linked to anomalies in FCG behavior at low stress intensity factor ranges, ΔK (below the conventional ΔK_th_), is reviewed and discussed based on findings from several alloys reported in the literature. The standard application of ΔK in FCG analysis assumes that da/dN decreases consistently with a reduction in the mechanical ΔK and approaches the threshold value ΔK_th_ when da/dN is ≤10^−7^ to 10^−8^ mm/cycle. However, some materials do not exhibit a consistent decrease in the FCG rate with a reduction in ΔK as the applied mechanical driving force. This uncommon da/dN versus ΔK behavior is not fully understood and cannot be explained solely by crack closure, creep, or metallurgical factors. Understanding and addressing this unexplained FCG behavior and its underlying physics pose a significant challenge for scientists and engineers. Currently, a lack of awareness of this phenomenon or its oversight in damage-tolerant design creates safety issues and may lead to non-conservative fatigue life predictions for structures subjected to service loads with high K_max_ and low ΔK values, such as those caused by high-frequency vibrations.

## 2. Anomalies in FCG Rates at Low ΔK

**Figure 4:**
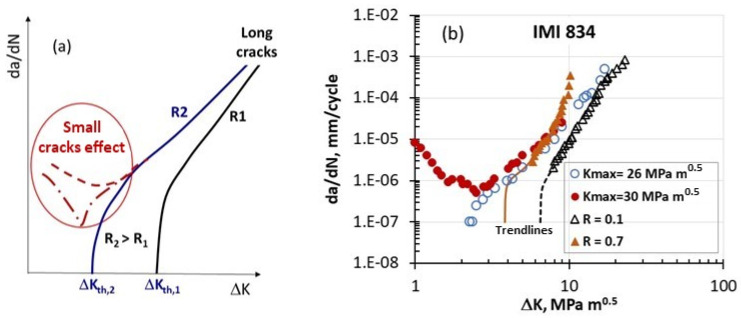
(**a**) A schematic illustration of long and small FCG behavior, and (**b**) Marci’s data [15] for long cracks tested under constant-ΔK and constant-K_max_ conditions. At a constant K_max_ = 30 MPa√m, the data show a dip followed by an increase in da/dN.

It is well known that small cracks can propagate below the ΔK_th_ of longer cracks. This behavior is often regarded as an anomaly in small-crack growth, based on the understanding that small cracks are less influenced by plasticity-induced crack closure (PICC) due to the relatively small size of the crack wake. Subsequently, as small cracks increase in size, the development of a wake and associated PICC causes their behavior to resemble that of longer cracks, as illustrated in Figure 4a. This PICC explanation has been debated for the past 50 years [14]. Interestingly, similar atypical FCG behavior has been documented in the literature for long cracks in the low-ΔK region [15,16,17,18,19,20,21,22,23,24,25,26,27,28]. The following sections show experimental results taken from the literature on this unusual behavior in several alloys, including IMI834 and Ti-6246 (wavy slip), Al 8009 (partially planar slip), 7075-T651 (mixed planar and wavy slip), and S690QL steel (wavy slip). Interestingly, some nickel-based alloys reported in the literature, such as Ni100, Inconel 718, and Rene’95, which exhibit planar slip when tested at room temperature, do not display this unusual behavior.

### 2.1. Behavior of Some Titanium-Based Superalloys

In 1996, Marci was the first to report [15] that, under constant-K_max_ testing conditions, a long crack displays an unusual FCG rate at ΔK < ΔK_th_, resembling the behavior of small cracks (Figure 4a). Figure 4b depicts the typical trendlines along with this unexpected behavior for long cracks in the IMI 834 titanium alloy [15].

This unusual behavior frequently aligns with FCG rates at the knee point between the near-threshold and Paris regions, which is approximately 10^−6^ mm/cycle. Marci observed [15] that, for the IMI 834 alloy investigated, this unusual FCG behavior begins when the K_max_ value exceeds 26 MPa√m. On the other hand, for the K_max_-controlled test with K_max_ < 26 MPa√m, a characteristic continuous reduction in da/dN is observed as ΔK decreases. In his second and last 1997 paper on this topic [16], Marci described and discussed his observations of unusual FCG behavior in three Ti alloys: IMI834, IMI 685, and Ti-6Al-6V-2Sn. In his 1997 paper, Marci stated that “It is a well-known fact in the turbine industry that some disk materials, developed and tested according to the *state of the art* technology, exhibit poor performance in service.” In the same paper, he also stated, “One can check the possibility whether this abnormal crack growth behavior could be responsible for the poor service performance of the Ti alloys”. These statements and the reported experimental observations on abnormal FCG behavior were disturbing and viewed as inconvenient, leading to Marci being cut off from further financial support for his research and publications in this area [29]. To date, the abnormal behavior of long fatigue crack growth (FCG) in some materials, observed more prevalently in K_max_-constant tests than in R-constant tests, has not been explained by either metallurgical analysis or failure mechanism studies.

It can be noted that, to date, no plausible model or mechanism exists in the literature that can explain the Marci effect. Some questions that need to be asked include the following: Does this unusual behavior of long cracks below ΔKth relate to a similar phenomenon observed for small/short cracks? If they are related, can they be explained using the same mechanisms? According to our current understanding, design engineers utilize the FCG threshold concept, which postulates that if ΔK is below ΔK_th_ (or ΔK_th,eff_), significant FCG does not occur. However, if cracks can grow with a da/dN rate larger than 10^−7^ mm/cycle even when the applied ΔK < ΔK_th_, the traditional design approach that relies on a long-crack threshold, ΔK_th_, may be non-conservative and pose a risk.

**Figure 5:**
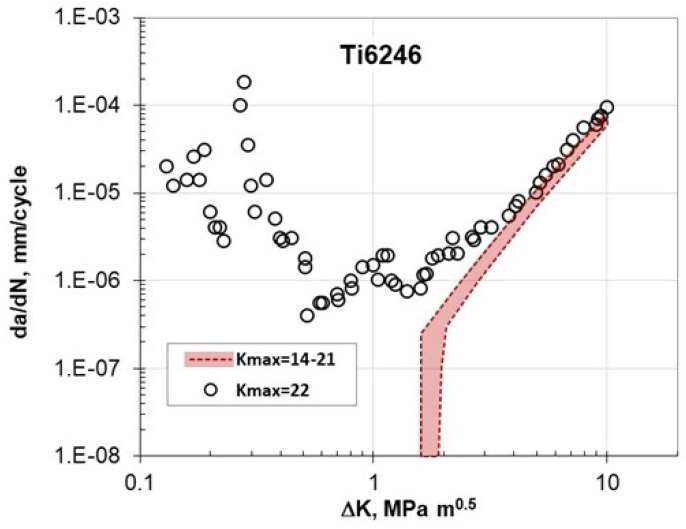
da/dN-ΔK data for Ti-6246 in different K_max_-constant tests [17].

Shortly after Marci’s publications, in 1998, Lang et al. [17] investigated two superalloys, Ni100 and Ti-6246, under constant-K_max_ testing conditions. They found that the Marci effect was not observed in the Ni-100 alloy. Conversely, the Ti-6246 alloy exhibited an unusual crack growth effect, classified as Type I. This unusual FCG behavior was evident for K_max_ ≥ 22 MPa√m, as depicted in Figure 5. It can be noted that K_max_ = 22 MPa√m is about 72% of the fracture toughness, K_IC_, for the Ti alloy investigated.

They suggested that the impact of room-temperature creep is minimal, as no differences were observed between 10 Hz and 50 Hz frequencies.

**Figure 6:**
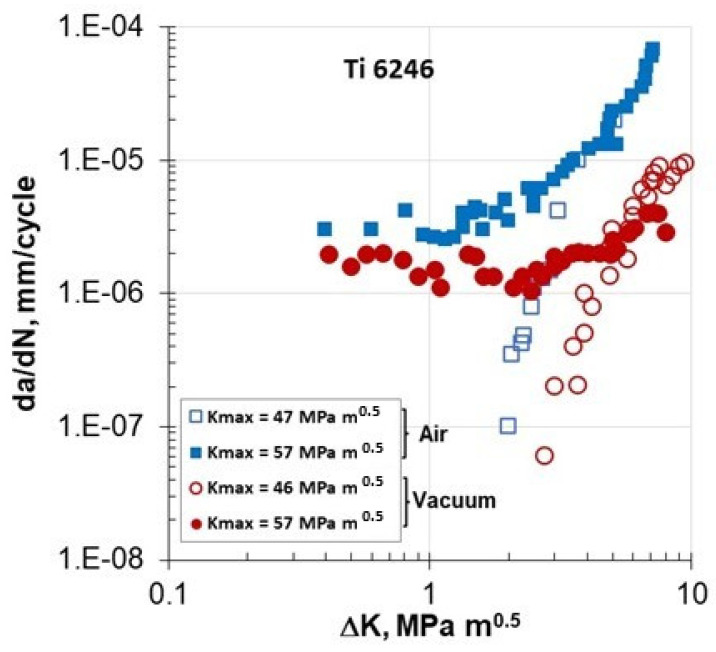
da/dN-ΔK data for the Ti-6246 alloy in laboratory air and vacuum from K_max_-constant tests [18].

Four years after Marci’s first paper [15], Sarrazin-Baudoux et al. [18], in 2000, presented their findings on the FCG rates of the Ti-6246 alloy at room temperature in laboratory conditions and in a vacuum of 10^−4^ Pa. The results from Petit et al. are shown in Figure 6 for K_max_ levels of 46–47 MPa√m and 57 MPa√m. At the 46–47 MPa√m level, the FCG in both the air and vacuum environments exhibited typical threshold behavior. However, at a higher K_max_ = 57 MPa√m, unexpected Type I FCG behavior was observed at a low stress intensity factor range of ΔK < ΔKth ≅ 2 MPa√m in both environments. The observed behavior was considered an intrinsic mechanism, given that it was seen in both environments. It is noteworthy that the rate of fatigue crack growth in air was approximately three times faster than in the vacuum, suggesting that the presence of air may facilitate the cracking process. Furthermore, the test conducted at 57 MPa√m was performed at two different frequencies: 3.5 Hz and 35 Hz. For ΔK values equal to or greater than 3 MPa√m, there was no observed effect of frequency on FCG behavior. However, for ΔK values below 2 MPa√m, the crack growth rate (da/dN) was higher at 3.5 Hz compared to 35 Hz. When the data from both frequencies were plotted in terms of da/dt [18] (though not shown here), the results indicated that the frequency effect almost disappeared.

Petit et al. [18] implied that the accelerated FCG behavior observed for ΔK values below 2 MPa√m was possibly influenced by ambient-temperature creep.

### 2.2. Unusual FCG in Aluminum Alloys

**Figure 7:**
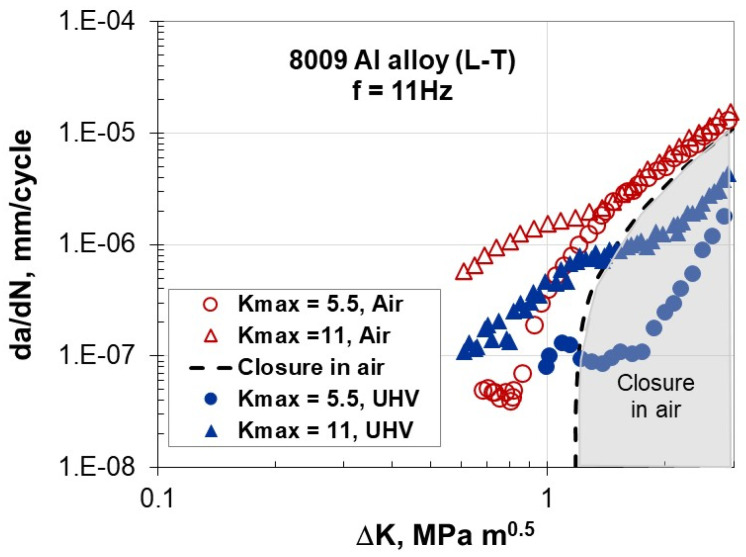
Near-threshold FCG behavior for K_max_-controlled tests at 5.5 and 11 MPa$\sqrt{m}$ in laboratory air and UHV [19].

FCG data for the 8009 aluminum alloy [19] in a lab environment and an ultra-high vacuum (UHV ~10^−6^ Pa) are depicted in Figure 7. To avoid the effect of crack closure at low ΔK values near the threshold, the tests were conducted under constant K_max_ at 5.5 and 11 MPa√m. Type III unusual FCG behavior was particularly observed at low ΔK, corresponding to the closure-free region (Figure 7). In this region, the da/dN rates in the UHV were approximately five times slower than those in laboratory air, indicating the impact of the air environment on the crack-tip damage process. Careful microscopic examination of the fracture surface carried out just after the transition to accelerated da/dN in air (at ΔK = 0.8 MPa√m for K_max_ = 5.5 MPa√m and ΔK = 1.3 MPa√m for K_max_ = 11 MPa√m) revealed a micro-void morphology analogous to elevated-temperature creep crack growth [19]. It was found that the region corresponding to accelerated FCG behavior, along with associated void formation, was confined to the specimen’s center (corresponding to plane strain), which promoted crack tunneling. The transition to slant cracking was observed near the threshold, leading to an accelerated FCG rate as ΔK decreased. It was also reported that when the test results of da/dN for different frequencies were converted to da/dt–ΔK plots, the frequency effect nearly disappeared. The unusual FCG rate at low ΔK was attributed to time-dependent crack-tip damage.

On the other hand, the two Ni-based superalloys 718 and Rene’95 did not exhibit the Marci effect, which coincides with the observations of Lang et al. [17] for a single-crystal Ni-based superalloy.

**Figure 8:**
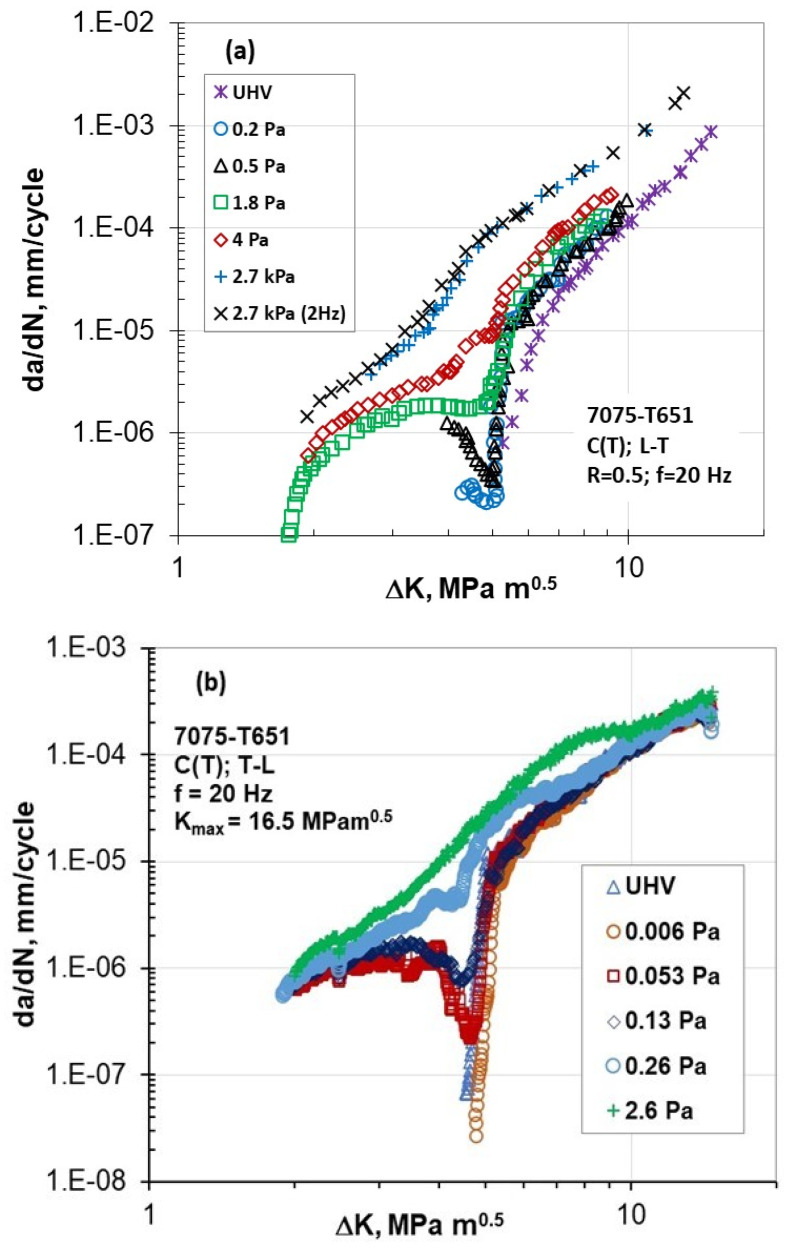
Unusual FCG of 7075-T651 Al alloy at high vacuum pressure and different water vapor pressures in (**a**) R-constant and (**b**) K_max_-constant tests [21,22].

Recently, Burns et al. [21] and Jones [22] reported unexpected Type II behavior in da/dN versus ΔK for the 7075-T6 aluminum alloy (L-T orientation) under constant R = 0.5 conditions (Figure 8a) and in constant K_max_ = 16.5 MPa√m tests (Figure 8b) (T-L orientation).

Three environments were used: laboratory air, different water vapor pressures, and UHV (~10^−6^ Pa). The FCG behavior data in the lab air and UHV do not show the Marci effect. Instead, they exhibit monotonically decreasing da/dN-ΔK curves, each trending towards different thresholds. The effect of water vapor pressure on near-threshold crack growth rates is evident under both R-constant and K_max_-constant test conditions.

In order to examine the effect of frequency at a pressure of 2.7 kPa, tests at R = 0.5 were conducted at two frequencies: 2 Hz and 20 Hz. Despite an order of magnitude difference in frequencies, overlapping da/dN-ΔK curves were obtained. An examination of the results depicted in Figure 8 indicates that at low moisture content and ΔK around 5 MPa√m, a sharp dip and rebound in FCG rate behavior is observed. With increased moisture content, this effect gradually vanishes, and the FCG rate monotonically approaches a threshold below 2 MPa√m.

### 2.3. Unusual FCG Behavior in Steel

**Figure 9:**
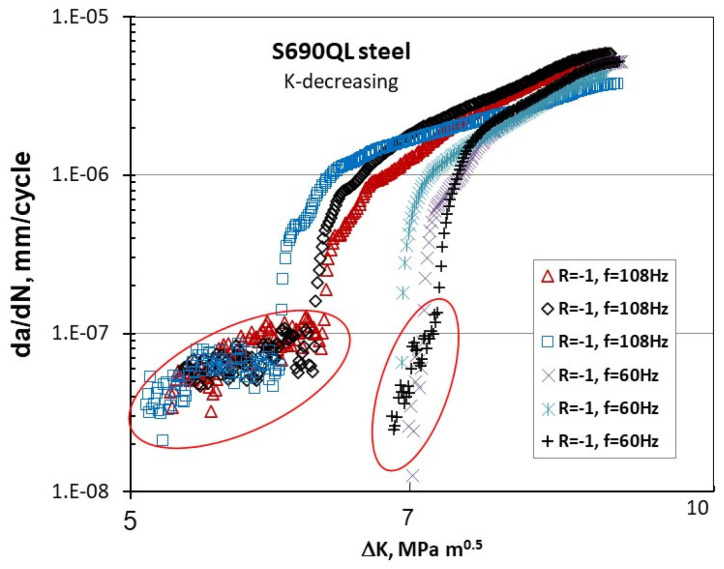
Effect of frequency on the da/dN-ΔK behavior in S690QL steel [23] (with a yield stress of 810 MPa) at R = −1.

Figure 9 depicts the da/dN-ΔK behavior of S690QL steel [23] at a negative R value of −1, tested at two frequencies: 108 Hz and 60 Hz.

**Figure 10:**
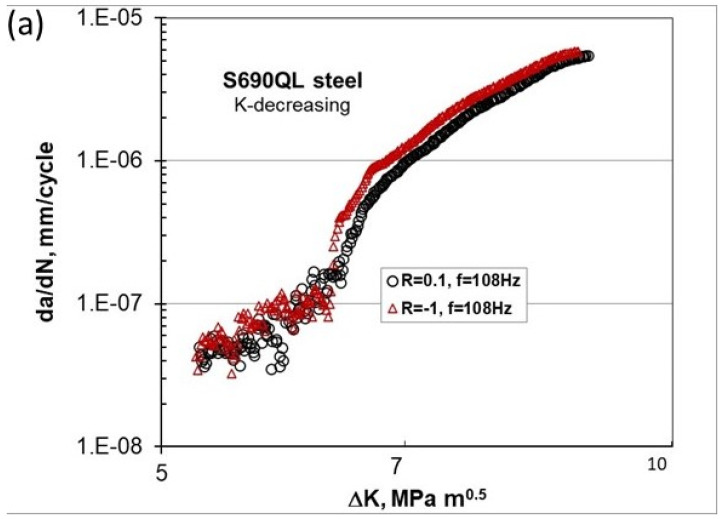

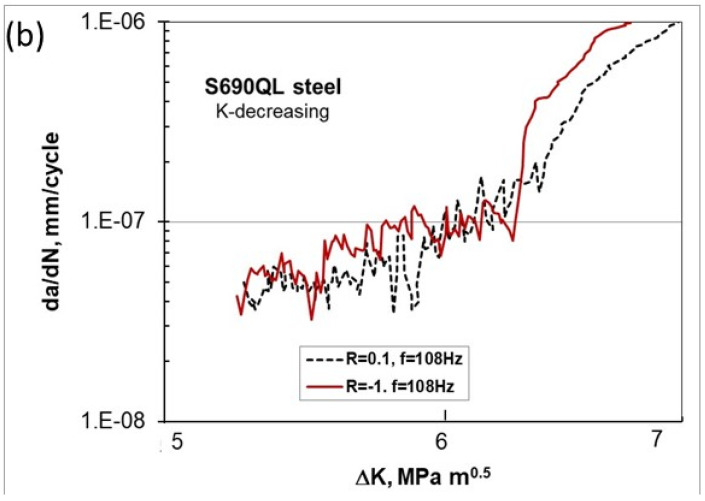
FCG behavior of S690QL steel near the threshold at R = 0.1 and −1 and 108 Hz (**a**) with a coarse scale and (**b**) a refined scale [23].

An unusual near-threshold behavior (Type III) is observed below 10^−7^ mm/cycle, suggesting that using a ΔK_th_ value based on an FCG rate of 10^−8^ mm/cycle would be non-conservative. The Type III crack propagation below 10^−7^ mm/cycle exhibits significantly greater scatter and variation compared to the rates above 10^−7^ mm/cycle. A similar scatter/variation in Type III FCG behavior at da/dN < 2 × 10^−7^ mm/cycle is shown in Figure 10a for S690QL at the threshold and near the threshold for two R-ratios of 0.1 and −1 obtained at 108 Hz.

The unusual FCG behavior for da/dN < 2 × 10^−7^ mm/cycle is practically independent of whether the R-ratio is positive or negative and is contained within the same scatter. Figure 10b shows, in more detail, the FCG behavior for rates lower than 2 × 10^−7^ mm/cycle. It is seen that this unusual FCG behavior (below 2 × 10^−7^ mm/cycle) exhibits up-and-down or staircase-type behavior [23]. It can be noted that the unusual FCG behavior for these high-strength steels starts below 10^−7^ mm/cycle, which is about one order of magnitude lower than for Ti and Al alloys.

**Figure 11:**
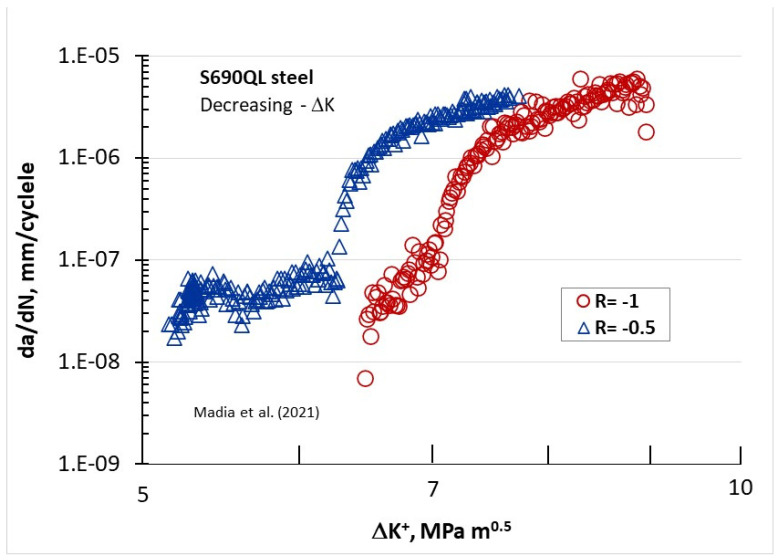
The da/dN behavior of S690QL steel [24] in terms of the positive part of ΔK^+^ at two negative R-ratios: −0.5 and −1.

Figure 11 depicts the da/dN behavior of S690QL steel [24], plotted against the positive portion of ΔK^+^ for two negative R-ratios: −0.5 and −1. Unusual FCG behavior is clearly seen at rates lower than 2 × 10^−7^ mm/cycle and is more pronounced at an R-ratio of −0.5 than −1. The limited steel data shown in Figure 9, Figure 10 and Figure 11 indicate that at very low ΔK, no FCG acceleration is observed, but the rate tends to plateau with decreasing ΔK.

S690QL steel was also tested near the threshold in laboratory air at two relative humidity (RH) levels of 30% and 70% [24]. An abrupt change in relative humidity resulted in transient FCG behavior, indicating an environmental effect on crack-tip damage and associated FCG behavior. Further testing is needed to clarify the role of humidity at very low ΔK close to or below the threshold.

## 3. Discussion

**Figure 12:**
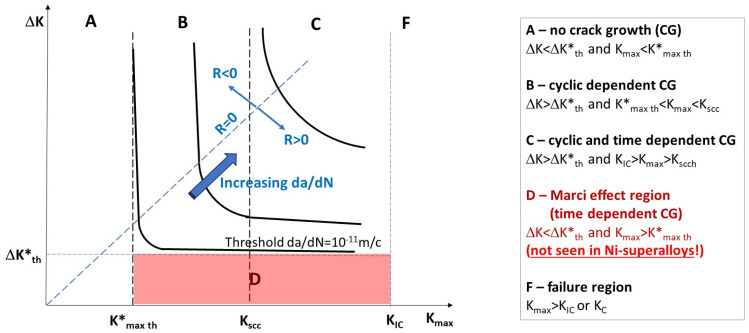
Illustration of hyperbolic da/dN = constant curves in terms of applied ΔK and K_max_ loading parameters and different regions of FCG behavior.

It is well recognized that the quantification and analysis of fatigue damage require two loading parameters: amplitude (or range) and mean (or maximum) values [30]. In the conventional analysis of high and low cycle fatigue, this is identified as the mean stress effect. In the case of HCF, the stress-based S-N approach is commonly used, where these two loading parameters are the stress amplitude, σ_a_, and the mean stress, σ_m_. Another well-known fatigue damage driving force, the so-called SWT parameter (SWT = $\sqrt{\sigma_{a}\sigma_{max}}$) proposed by Smith, Watson, and Topper, is widely used to account for the mean stress effect in both the LCF and HCF regions. On the other hand, traditional FCG analysis mostly uses the stress intensity factor range, ΔK, and load ratio, R, where R is not a loading parameter but a nondimensional measure of the K_min_/K_max_ ratio. Hence, for the last 50 years, ΔK and R have dominated FCG analysis since Elber [10] postulated that the load ratio’s effects on FCG are caused by an extrinsic factor called crack closure. However, when both S-N data and FCG data are represented in terms of a constant lifetime, N_f_, or a constant da/dN rate, they result in hyperbolic behavior [30]. Figure 12 illustrates the hyperbolic curves corresponding to a constant da/dN rate in terms of the applied ΔK and K_max_ loading parameters. It can be noted that as da/dN decreases, a sharper transition from the R > 0 region to the R < 0 region is observed (Figure 12). This sharp transition is particularly noticeable at the threshold due to the very limited plasticity involved in FCG propagation in the near-threshold region [30].

**Figure 13:**
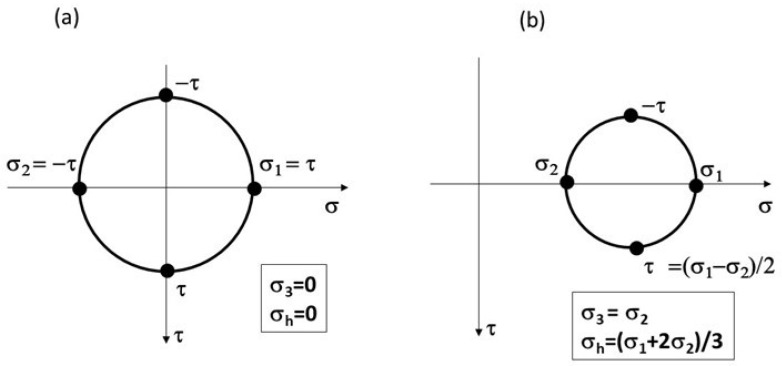
Illustration of principal and hydrostatic stresses: (**a**) planar slip and (**b**) wavy slip deformation.

Five different regions for possible FCG behavior are also indicated in Figure 12. Region D is associated with time-dependent unusual FCG behavior, which showed tunneling (plane strain) crack propagation with some influence from the environment. As was mentioned before, Lang et al. [17] and Newman and Piascik [20] did not observe the Marci effect in Ni-based superalloys, which are characterized by pronounced planar slip due to a possible low hydrostatic stress effect, σ_h_, on the gliding plane (Figure 13). The data presented indicate that the Marci effect is related to time-dependent void formation (due to cross-slip) in plane strain conditions for wavy slip alloys influenced by relatively high hydrostatic stress, σ_h_. Figure 13 shows a hypothetical state of hydrostatic stress (σ_h_) ahead of the fatigue crack tip under plane strain conditions for planar versus wavy slip alloys.

In addition to hydrostatic stress, the Marci effect is influenced by the R-ratio and is amplified by environmental factors. An active environment can increase cross-slip activity and void formation, leading to faster fatigue crack growth in laboratory air conditions compared to the inert environment of a vacuum (Figure 6 and Figure 7).

## 4. Conclusions

The established application of fracture mechanics to FCG analysis assumes that the crack growth rate, da/dN, decreases monotonically with decreasing ΔK and approaches the threshold value of ΔK_th_ with da/dN ≤ 10^−7^ mm/cycle. This commonly used assumption breaks down for certain Ti, Al, and steel alloys, which show a dip followed by a plateau or even an acceleration in FCG rates when tested under both K_max_- and R-ratio-controlled conditions at low ΔK ≤ ΔK_th_. Such unexpected FCG behavior is sensitive to the material/environment system, frequency, and slip deformation characteristics. The observed anomalies are not fully understood and cannot be accounted for by plasticity, roughness, or oxide-induced crack closure (PICC, RICC, or OICC) mechanisms. Understanding this anomalous behavior poses a significant challenge to scientists and engineers. Neglecting or disregarding it could jeopardize the fatigue safety of structures subjected to high mean service loads and low-amplitude vibrating conditions.

Therefore, the primary aim of this article is to inspire the fatigue research community to further investigate this uncommon FCG behavior at low ΔK values below ΔK_th_ for specific engineering alloys. Ultimately, future progress in this area depends on a deeper physical understanding of intrinsic mechanical deformation processes at very low ΔK values.

## Data Availability

The original contributions presented in this study are included in the article. Further inquiries can be directed to the corresponding author.
